# The Epidemiological Pattern of Skin Cancer from 2011 to 2022 among the Population of the Aseer Region, Kingdom of Saudi Arabia

**DOI:** 10.3390/cancers15184612

**Published:** 2023-09-18

**Authors:** Abdullah Mohammed Algarni, Hamza Salim Alshehri, Ahmed Saad Al Zomia, Mohammed Abdulrahman Alhifthi, Lama Ali Lahiq, Faisal Mohammed Al Fae, Awad Mohammed Alwadie, Shuruq Abdullah Al-Qahtani, Faisal Suhaim Al Amri, Faisal Hassan Tobeigei

**Affiliations:** 1 Aseer Central Hospital, Ministry of Health, Abha 62523, Saudi Arabia; abaid1406@gmail.com (A.M.A.); hsalshehri@moh.gov.sa (H.S.A.); 2 Faculty of Medicine, King Khalid University, Abha 62529, Saudi Arabia; mohammed.alhifthii@gmail.com (M.A.A.); x.xlamooxx@hotmail.com (L.A.L.); dr.faisalfaye11@gmail.com (F.M.A.F.); awad.moh3347@gmail.com (A.M.A.); shorg2014@gmail.com (S.A.A.-Q.); faisal_s_h@hotmail.com (F.S.A.A.); 3 Department of Dermatology, College of Medicine, King Khalid University, Abha 62529, Saudi Arabia; ftobeigei@kku.edu.sa

**Keywords:** Saudia Arabia, skin cancer, malignant melanoma, basal cell carcinoma, squamous cell carcinoma

## Abstract

**Simple Summary:**

We found that the incidence of skin cancer in the Saudi population of the Aseer Region of the country has varied over the years, with an overall risk of 9.9% of developing cancer before the age of 75 years. The most common types of skin cancer were squamous cell carcinoma and basal cell carcinoma. The head and neck region were the most affected areas, and the majority of cases were observed in individuals aged 61–80 years. There was a higher incidence of skin cancer in men compared with women, and the dataset primarily consisted of Saudi nationals.

**Abstract:**

The overall risk of developing cancer before the age of 75 years in the Kingdom of Saudi Arabia is 9.9%. We aimed to explore the pattern of skin cancer, specifically among the Saudi population residing in the Aseer region. We obtained data from the medical records of Aseer Central Hospital regional histopathological laboratory considering surgical pathology reports from 2011 to 2021. The 61–80-year-old age group represented most of the cases (41.4%), followed by the 41–60-year-old group at 24.1%. Men made up the majority of the cases (59.4%). Furthermore, the dataset predominantly consisted of Saudi nationals (94.3% of the sample). The percentage of cases diagnosed each year relative to the cumulative number of skin cancer cases varied each year, ranging from 1.6% in 2011 to 11.6% in 2017. The most common diagnoses were squamous cell carcinoma (SCC) with 230 cases (41.1%) and basal cell carcinoma (BCC) with 147 cases (26.3%). The majority of cases occurred in the head and neck region (55.4%), followed by the lower limb (16.6%), trunk (13.6%), upper limb (8.2%), and pelvis (2.3%). There was a significant variation in the type of skin cancer across the age groups (*p* < 0.001) and across different body parts (*p* < 0.001). The incidence of skin cancer exhibited variability throughout the study period. The predominant diagnoses observed were SSC and BCC. Among the affected areas, the head and neck region displayed the highest prevalence, followed by the lower limb, trunk, upper limb, and pelvis.

## 1. Introduction

The skin is composed of two main layers: the outermost layer, known as the epidermis, and the underlying dermis. The epidermis contains various cell types, including keratinocytes (90%), melanocytes (8%), Merkel cells (1–2%), and Langerhans cells (1–2%) [1]. Any deviation or abnormality within this layer can result in different types of skin damage, including the development of cancer. The occurrence, impact on health, and death rates associated with skin cancer are consistently on the rise throughout the world [2].

Skin cancer poses a significant global public health problem, and its prevalence is steadily increasing. This trend has far-reaching consequences, impacting both the global economy and the workforce [3,4]. Broadly speaking, skin cancer is categorised into two main types: melanoma, which originates from dysfunctional melanocytes, and non-melanoma skin cancers (NMSCs), which develop from cells derived from the epidermis [5]. Melanoma arises from the abnormal proliferation of human melanocytes, which are pigment-containing cells found in varying proportions in different parts of the body. Melanocytes make up approximately 90% of the cells in the skin, 5% in the eyes, and 1% in the intestine [6]. Compared with other types of skin malignancies, melanoma represents only 1% of all skin tumours. However, it is important to note that despite advancements in therapeutic strategies, melanoma remains the most aggressive form of skin cancer. Its 5-year survival rate is typically low, ranging from 15% to 20% [7,8].

NMSCs are primarily categorised into two main subtypes: cutaneous squamous cell carcinoma (SCC) and basal cell carcinoma (BCC). These two subtypes alone account for approximately 99% of all NMSCs [9]. Multiple studies have indicated a notable annual increase in the incidence of NMSC throughout the world, ranging from 3% to 8%, since 1960. Furthermore, the incidence of NMSC is significantly higher compared with melanoma, with estimates suggesting it is approximately 18–20 times more prevalent [10]. Men are considered to be at a higher risk for NMSCs compared with women [11]. The inclination of women towards sun exposure and tanning [12] contrasts with the relatively lower likelihood of men adopting preventive behaviours [13] or detecting melanomas on their own [14].

The increasing incidence rates of skin cancer can be attributed to various factors acting in combination. These factors include heightened exposure to ultraviolet (UV) light or sunlight, increased engagement in outdoor activities, changes in clothing preferences, longer lifespans, depletion of the ozone layer, genetic predisposition, and, in certain cases, immunosuppression. BCC has been linked to intense UV exposure during childhood and adolescence, while chronic UV exposure during earlier decades is often associated with the development of SCC. Based on the increasing prevalence of skin cancers and the challenges in efficient drug delivery systems, it is necessary to develop new ways to prevent or cure the disease [15].

In 2020, a total of 27,885 cancer cases were diagnosed in the Kingdom of Saudi Arabia (KSA). Among these cases, 14,253 were men and 13,632 were women. Tragically, there were 13,069 deaths attributed to cancer during the same period. The overall risk of developing cancer before the age of 75 years in KSA stands at 9.9%. Specifically, the risk is 9.1% for men and 11.4% for women [16]. The most prevalent cancers in Saudi Arabia are breast cancer, colorectal cancer (CRC), prostate cancer, brain/central nervous system cancer, Hodgkin and non-Hodgkin lymphoma, kidney cancer, and thyroid cancer [17]. Regarding skin cancer in KSA, a study carried out in the south-west part of the country in 2004 indicated that BCC constituted 41% of all cancer cases, followed by SCC at 29%, Kaposi sarcoma at 18%, and malignant melanoma at 4% [18]. A retrospective study spanning 20 years among Saudi patients in the Eastern Region yielded similar findings [19]. Furthermore, in the Western Region, research conducted between 2000 and 2010 revealed BCC as the most prevalent cancer type, followed by SCC and malignant melanoma [20]. In the Southern Region, an investigation conducted from 1987 to 1991 involving 137 patients with skin cancer indicated that SCC was the predominant cancer type (41.6%), followed by BCC (36.5%) and malignant melanoma (11.7%). The authors attributed the increase in SCC cases (29.8%) to secondary SCC originating from osteomyelitis skin sinuses, keloids, and traumatic scars [20].

The rationale for this study stems from the lack of research on the pattern of skin cancer specifically among the Saudi population residing in the Southern Region. With no recent existing studies addressing this aspect, we aimed to fill this knowledge gap by examining the epidemiological pattern of skin cancer in the Aseer Region. We utilised electronic medical records, which provide a valuable source of data for studying disease patterns and prevalence.

## 2. Materials and Methods

### 2.1. Study Setting and Design

Aseer, positioned in the south-western part of Saudi Arabia, is among the administrative regions of the country. It is defined by the coordinates, 17.25°19.50′ N and 50.00°41.50′ E. The city of Abha serves as the emirate’s central hub. Aseer is 81,000 km^2^, and the estimated population in this region is around 1,563,000 people. In this retrospective cross-sectional study, we extracted data from the medical records of Aseer Central Hospital (ACH) regional histopathological laboratory. The pathology laboratory at the ACH stands as the sole central facility for malignant skin tumour diagnostics. It is entrusted with evaluating pathology specimens from various peripheral hospitals under the Ministry of Health in this region. Consequently, this comprehensive arrangement ensured the inclusion of all cases of malignant skin tumours, whether biopsied or excised, in this study. We conducted a thorough search within the surgical pathology reports covering the period from 2011 to 2021.

### 2.2. Study Population

We considered patients who had received a skin cancer diagnosis after a skin biopsy. We included all cases of skin cancer, regardless of the age, sex, or nationality of the patients. We only considered a single biopsy for each patient, even though there might have been multiple biopsies for the same patient. The skin biopsies were obtained from patients seen at the ACH, along with those from general hospitals situated in Khamis Mushayt, Tathleeth, Bisha, Zahran Al-Janoub, Sarat Abidah, Ahad Rufidah, Rigal Alma, and Muhayl. We excluded individuals with benign cutaneous conditions and other forms of cutaneous malignancies.

### 2.3. Data Collection

We searched the medical records by using a combination of free text search and an institutional code for malignancy to identify skin cancer cases. Trained specialists at the ACH were responsible for collecting and reviewing the data. Two dermatology experts categorised the histopathological diagnoses into predefined skin disease categories based on the *International Classification of Diseases, Tenth Revision* (ICD-10) and *Dermatology* (Bolognia) textbook. They also assessed the study’s outcome.

During the manual review, we extracted specific information from the reports, such as biopsy date, medical record number, age, sex, nationality, pathological diagnosis, and tumour location and recorded it in an Airtable data sheet. Moreover, we collected additional parameters related to the nature of the lesion, including its type, size, site, borders, depth, and lymphovascular invasion. To ensure data accuracy, we performed manual checks to identify and remove any duplicates. Subsequently, we exported the data to Microsoft Excel to facilitate further analysis.

### 2.4. Statistical Analysis

We conducted statistical analyses with SPSS Statistics version 26.0. The categorical variables are presented as the total number and percentage, and normally distributed continuous variables with a normal distribution are presented as the mean and standard deviation. We used the chi-square test to examine associations between categorical variables. When the assumptions of this test were not met, we used the Monte Carlo test as an alternative. We considered *p* < 0.05 to indicate statistical significance. We used the line chart function of Microsoft Excel to illustrate the overall trend of skin cancer from 2011 to 2021. The denominator represents the total number of skin cancer diagnoses from 2011 to 2021, while the numerator denotes the number of cases diagnosed annually within the same period.

### 2.5. Ethical Approval

The study was granted approval by the Aseer Institutional Review Board under the Ministry of Health, identified by reference number H-06-B-091. As the study involved the use of de-identified and anonymised data extracted from medical records, it was conducted in accordance with ethical norms. Moreover, the research followed the principles outlined in the Declaration of Helsinki. The utmost confidentiality of patient data was maintained, with access limited exclusively to the principal investigator and the designated statistician.

## 3. Results

The mean age of the patients with skin cancer was 63.4 ± 21.3 years. Based on the age distribution, most of the patients were in the 61–80-year age range (41.4%), followed by the 41–60-year age range (24.1%). The percentage of individuals below 40 years and above 80 years was 15.4% and 19.1%, respectively. In terms of sex, the majority of patients were men (59.4%); women accounted for 40.6% of the cases. Furthermore, the dataset predominantly consisted of Saudi nationals (94.3% of the sample). The majority of patients (87.0%) were from the ACH; 11.3% of patients were obtained from other sources, while a small proportion of 1.8% had an unknown source. Table 1 describes the presentation of skin cancer cases. A large percentage of cases (44.8%) had skin lesions (251 cases, 44.8%), while ulcers were present in 20.9% of cases. Only 1.4% of cases were treated with immunosuppressive therapy.

**Figure 1:**
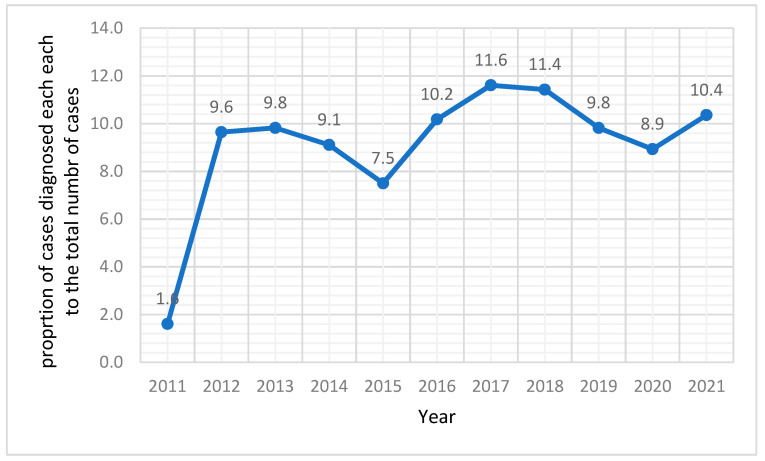
Trends in percentage of skin cancer diagnoses from 2011 to 2021 in the Aseer region, Kingdom of Saudia Arabia.

Figure 1 displays the percentage of cases diagnosed with skin cancer for each year from 2011 to 2021 relative to the total number of diagnosed cases. There have been some fluctuations in the percentages over the years, indicating variations in the yearly incidence of skin cancer. The percentages range from a low of 1.6% in 2011 to a high of 11.6% in 2017. Overall, the data suggest a somewhat consistent occurrence of skin cancer cases, with some notable peaks and dips throughout the years.

Table 2 highlights the distribution of different skin cancers within specific age groups. The most common diagnoses were SCC (41.1%) and BCC (26.3%). There were fewer cases for other types such as adnexal neoplasms, B-cell lymphoma of the skin (BCLS), cutaneous T-cell lymphoma (CTCL), cutaneous metastases, cutaneous neural neoplasms, fibrous/fibro histiocytic neoplasms, mastocytosis, melanoma, and vascular neoplasms. Table 3 further highlights the distribution of skin cancer across different locations on the body. SCC and BCC were prominent in all age ranges. There were significant variations in the type of skin cancer across the age groups (*p* < 0.001). It is worth noting that there was not a significant difference concerning sex and the type of skin cancer.

Table 3 shows the distribution of skin cancer in different body parts. The majority of cases occurred in the head and neck region (57.3%). Other significant locations include the lower limb (16.6%), the trunk (14.8%), the upper limb (8.6%), and the pelvis 13 (2.3%). There was a significant difference in the distribution of skin cancer diagnosis across different body parts (*p* < 0.001).

**Figure 2:**
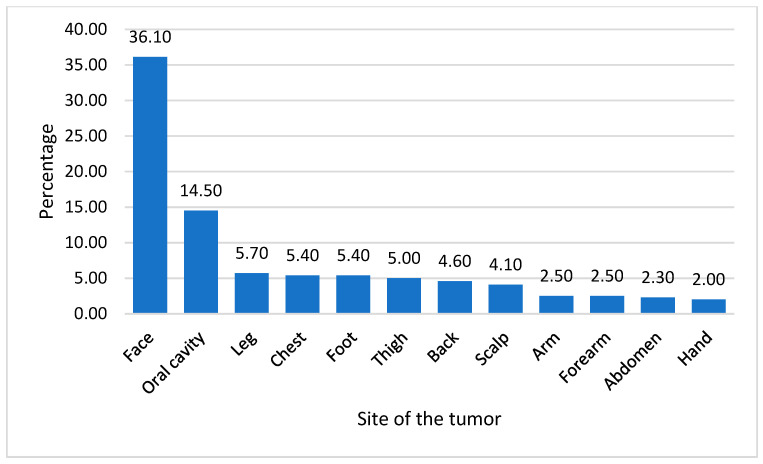
Distribution of skin cancers cases in body parts from 2011–2021 in the Aseer Region, Kingdom of Saudi Arabia.

Figure 2 provides insight into the specific parts of the body affected by skin cancer. The face was the most commonly affected (36.1%), followed by the oral cavity (14.5%), leg (5.7%), chest (5.4%), and foot (5.4%). Other affected areas include the thigh, back, scalp, arm, forearm, abdomen, and hand, each with a frequency ranging from 2.0% to 5.7%.

As shown in Table 4, SCC was the most prevalent form of skin cancer, with invasive SCC constituting 25.7% of cases. BCC was a close second, with nodular BCC accounting for 25.2% of cases. Mycosis fungoides (MF) dominated the CTCL category at 52%, while Kaposi sarcoma was the most common type of vascular neoplasms (36%). Breast-origin cutaneous metastasis represented 13% of cases. Nodular melanoma was the primary malignant melanoma subtype (3.2%).

**Figure 3:**
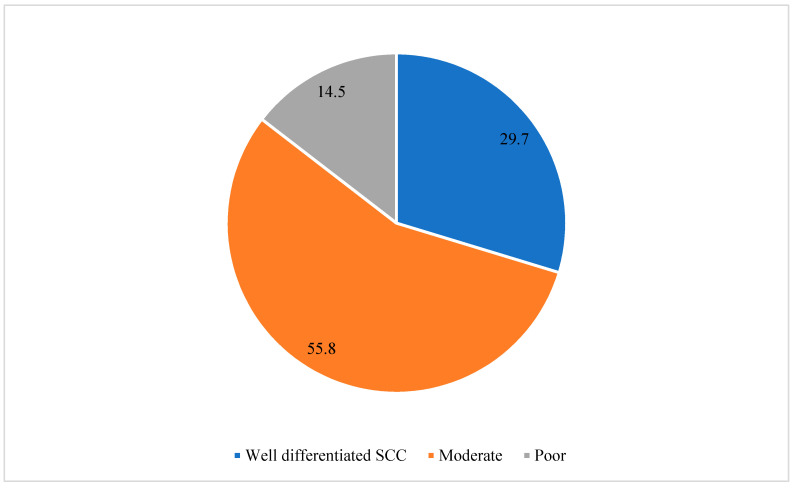
Distribution of squamous cell carcinoma (SCC) differentiation grades from 2011 to 2021 in the Aseer Region, Kingdom of Saudi Arabia.

The majority of SCC cases were of moderate differentiation (55.8%). Well-differentiated SCC constituted 29.7% of cases, while poorly differentiated SCC represented 14.5% of the cases (Figure 3).

## 4. Discussion

Despite the relatively low incidence of skin cancer compared with light-skinned Caucasians, the Saudi population still faces a significant risk and the potential for higher morbidity and mortality due to late presentations resulting from a lack of awareness [19]. In this study, we aimed to outline the epidemiological trends in skin cancer in the Aseer region of KSA from 2011 to 2021. We utilised electronic medical records as a valuable source of data. We reviewed the data of 560 patients and found that the number of skin cancer cases diagnosed fluctuated year to year. SCC emerged as the most frequently diagnosed type, followed closely by BCC. For SCC, invasive SCC constituted the prevailing subtype. Among the less commonly diagnosed skin tumours, we observed vascular neoplasms, melanoma, and cutaneous metastasis. Our findings unveiled a significant correlation between age and the type of skin cancer, with a higher prevalence among adults aged > 60 years. However, our analysis did not indicate any association between skin cancer and sex. Moreover, we noted a link between the type of skin cancer and its distribution. Specifically, the head and neck region emerged as the most frequently affected area, followed by the lower limbs.

We found a low incidence of skin cancer in the Aseer region, which can be attributed to several factors. The Saudi population predominantly consists of individuals with olive skin and Fitzpatrick skin types III–V, which inherently provides some protection against DNA damage induced by sunlight, thereby reducing the risk of carcinogenesis [21]. Furthermore, cultural practices in the region, such as the widespread use of full body cover clothing among both men and women, serve as a sun-protective measure. The prevalence of women covering their faces as part of cultural norms may explain the observed male predominance in skin cancer cases. Although certain regions of KSA experience high sun exposure due to the dry climate, the UV radiation in these areas is not biologically active [21]. This observation could potentially account for the lack of a significant association between skin cancer and sex, in contrast to what has been reported in prior studies [22].

In this study, the most frequently diagnosed skin cancer types were SCC (41.7%), BCC (26.25%), vascular neoplasms (6.96%), cutaneous metastasis (5.36%), and melanoma (5.18%). The average age of the patients diagnosed with skin cancer was 63.4 ± 21.3 years. Interestingly, our findings parallel those of Bahamdan et al. [20], who conducted a similar study in the same setting in 1991. In that study, there were a total of 137 cases between 1987 and 1991. Just as in our study, SCC was the most prevalent type of skin cancer, comprising 41.6% of cases, followed by BCC at 36.5% and malignant melanoma at 11.7%. These comparative findings lend further weight to the consistency of the prevalence and stability of different skin cancer types over time in the region. Likewise, Al-Dawsari and Amra [19] analysed the skin cancer epidemiology among Saudi patients receiving care at the Johns Hopkins Aramco Healthcare Centre in Dhahran, in the Eastern Province of KSA. They reported that BCC (36%) was the most frequent cutaneous malignancy, followed by SCC (23%). The head and neck region emerged as the predominant site for both types of tumours. Furthermore, AlSalman et al. [23] reviewed medical records of patients with skin cancer between 2003 and 2016; they included a total of 593 cases in the analysis. Among these cases, 279 were diagnosed with NMSCs, the majority (95%) of which were BCC, SCC, or a combination of both in a few cases. Specifically, the frequency was 50.2% for BCC and 44.8% for SCC. Al-Dawsari and Amra [19] examined the records of 204 patients with skin cancer. Among these cases, the most common cutaneous malignancies were BCC (36%) followed by SCC (23%). Both types of tumours were predominantly located in the head and neck region. The third-most-common malignancy was MF at 11%. Malignant melanoma was the fourth-most-prevalent skin malignancy, accounting for 7% of cases, with the lower extremities being the most frequent location for these tumours. Mufti [24] analysed the skin cancer patterns among Saudi individuals attending King Abdulaziz University Hospital in Jeddah between January 2000 and December 2010. The researcher reported that 28.3% were diagnosed with BCC, 24.5% with SCC, 18% with MF, 10.3% with malignant melanoma, and 5.7% with other malignancies. The consistency of the proportions of particular skin cancer types like SCC, BCC, and malignant melanoma across multiple studies conducted at different times implies that there may be underlying factors influencing the consistent nature of these patterns. Possible factors include environmental circumstances, genetic predispositions, lifestyle elements, or healthcare approaches that collectively impact the distribution of skin cancer types within the population over extended durations [25].

In the present study, the head and neck region emerged as the predominant location for the majority of cases (57.3%). Specifically, this region represented 74.4% of SCC cases and 83.0% of BCC cases. Other significant locations include the lower limb, trunk, upper limb, and pelvis. Furthermore, the face was the most commonly affected area, followed by the oral cavity, leg, chest, and foot. The majority of cases presented skin lesions, followed by ulcers. These results align with findings from other studies conducted in Saudi Arabia [19,20].

It has been estimated that over 50% of all cancer diagnoses occur in individuals aged ≥ 65 years, and this proportion is projected to increase to approximately 70% by 2030 [26]. Our findings reveal a notable correlation between age and the occurrence of skin cancer. Particularly noteworthy is that nearly two thirds of diagnosed cases of SCC and BCC occurred in individuals > 40 years old. Age undeniably serves as a significant risk factor for skin cancer, and the number of cases will likely continue to rise with the increase in life expectancy [27,28,29].

### Strengths and Limitations

There are a number of significant limitations that should be acknowledged. First, the reliance on medical records for data retrieval introduces the risk of encountering incomplete or inconsistent information. This aspect has the potential to impact the accuracy and comprehensiveness of the findings. Furthermore, we did not investigate the follow-up and outcomes associated with the management of skin cancer cases. Consequently, our comprehension of the long-term effects and treatment outcomes remains constrained. Nevertheless, it is crucial to underscore that this study represents the first research conducted in the Aseer region of KSA, aimed at scrutinising the epidemiology of skin cancer. Notwithstanding these limitations, our study offers valuable insights into the occurrence and attributes of skin cancer within this area.

## 5. Conclusions

In summary, we found that the skin cancer incidence varied from 2011 to 2021 in the Aseer Region, KSA, ranging from 1.6% to 11.6%. The most common diagnoses were SSC and BCC. The head and neck region was the most affected area, followed by the lower limb, trunk, upper limb, and pelvis. The incidence of skin cancer was significantly associated with increasing age, but there was no significant association with sex. Moreover, the distribution of skin cancer types exhibited variation based on the affected body regions. These findings provide important insights into the epidemiology of skin cancer in the studied population. By describing the epidemiological pattern of skin cancer in this specific region, our study contributes to a better understanding of the disease’s incidence and characteristics. To promote the early detection and prevention of skin cancer, it is crucial to implement public awareness campaigns that can educate the general population about the risk factors, signs, and symptoms of skin cancer, as well as the importance of regular self-examinations and seeking medical attention for any suspicious skin lesions. Customised prevention strategies could concentrate on distinct age groups.

## Figures and Tables

**Table 1:** Age, sex, and nationality of patients diagnosed with skin cancer from 2011 to 2021 in the Aseer region, Kingdom of Saudi Arabia.

Variable		Number	%
Age range (years)	≤40	86	15.4
	41–60	135	24.1
	61–80	232	41.4
	>80	107	19.1
	mean ± SD	63.4 ± 21.3	
Sex	Male	57	59.4
	Female	39	40.6
Nationality	Saudi	528	94.3
	Non-Saudi	32	5.7
Source of the patient	Aseer Central Hospital	487	87.0
	Other	63	11.3
	Unknown	10	1.8
Skin cancer presentation	Skin lesion	251	44.8
	Ulcer	117	20.9
Use of immunosuppressive therapy	Yes	8	1.4

**Table 2:** Skin cancer across different age ranges from 2011 to 2021 in the Aseer Region, Kingdom of Saudi Arabia.

Type of Skin Cancer		Total	Age Range (Years)				*p*
			≤40	41–60	61–80	>80	
Squamous cell carcinoma	*n*	230	13	64	101	52	<0.001
	%	41.07%	5.70%	27.80%	43.90%	22.60%	
Basal cell carcinoma	*n*	147	10	28	71	38	
	%	26.25%	6.80%	19.00%	48.30%	25.90%	
Vascular neoplasms	*n*	39	7	15	15	2	
	%	6.96%	17.90%	38.50%	38.50%	5.10%	
Melanoma	*n*	29	4	3	15	7	
	%	5.18%	13.80%	10.30%	51.70%	24.10%	
Cutaneous metastases	*n*	30	5	11	10	4	
	%	5.36%	16.70%	36.70%	33.30%	13.30%	
Fibrous/fibrohistiocytic neoplasms	*n*	9	5	4	0	0	
	%	1.61%	55.60%	44.40%	0.00%	0.00%	
Adnexal neoplasms	*n*	4	0	0	3	1	
	%	0.71%	0.00%	0.00%	75.00%	25.00%	
Cutaneous neural neoplasms	*n*	3	1	0	2	0	
	%	0.54%	33.30%	0.00%	66.70%	0.00%	
Cutaneous T-cell lymphoma	*n*	60	37	9	11	3	
	%	10.71%	61.70%	15.00%	18.30%	5.00%	
B-cell lymphoma of the skin	*n*	3	0	0	3	0	
	%	0.54%	0.00%	0.00%	100.00%	0.00%	
Mastocytosis	*n*	6	4	1	1	0	
	%	1.07%	66.70%	16.70%	16.70%	0.00%	

**Table 3:** The distribution of skin cancer across different body parts from 2011 to 2021 in the Aseer Region, Kingdom of Saudi Arabia.

Type of Skin Cancer		Affected Body Part					*p*
		Head and Neck	Upper Limb	Trunk	Pelvis	Lower Limb	
Squamous cell carcinoma	*n*	171	16	15	9	19	<0.001
	%	74.30%	7.00%	6.50%	3.90%	8.30%	
Basal cell carcinoma	*n*	122	5	13	0	7	
	%	83.00%	3.40%	8.80%	0.00%	4.80%	
Vascular neoplasms	*n*	2	7	4	0	26	
	%	5.10%	17.90%	10.30%	0.00%	66.70%	
Melanoma	*n*	5	4	0	0	20	
	%	17.20%	13.80%	0.00%	0.00%	69.00%	
Cutaneous metastases	*n*	7	0	19	1	3	
	%	23.30%	0.00%	63.30%	3.30%	10.00%	
Fibrous/fibrohistiocytic neoplasms	*n*	2	4	1	0	2	
Adnexal neoplasms	%	22.20%	44.40%	11.10%	0.00%	22.20%	
	*n*	3	0	1	0	0	
Cutaneous neural neoplasms	%	75.00%	0.00%	25.00%	0.00%	0.00%	
	*n*	0	0	2	0	1	
	%	0.00%	0.00%	66.70%	0.00%	100.00%	
Cutaneous T-cell lymphoma	*n*	7	9	24	3	17	
	%	11.70%	15.00%	40.00%	5.00%	28.30%	
B-cell lymphoma of the skin	*n*	2	1	0	0	0	
	%	66.70%	33.30%	0.00%	0.00%	0.00%	
Mastocytosis	*n*	0	2	4	0	0	
	%	0.00%	33.30%	66.70%	0.00%	0.00%	
Total		321 (57.3%)	48 (8.6%)	83 (14.8%)	13 (2.3%)	95 (17.0%)	

**Table 4:** The distribution of the most common skin cancer types and subtypes from 2011 to 2021 in the Aseer Region, Kingdom of Saudi Arabia.

Type of Skin Cancer	Sub Type	*n*	%
Squamous cell carcinoma	Invasive squamous cell carcinoma	144	25.7
	Squamous cell carcinoma in situ (Bowen’s ds)	27	4.8
	Verrucous squamous cell carcinoma	7	1.3
	Unknown squamous cell carcinoma	45	8
Basal cell carcinoma	Nodular basal cell carcinoma	141	25.2
	Superficial basal cell carcinoma	2	0.4
	Morpheaform basal cell carcinoma	2	0.4
	Fibroepithelial basal cell carcinoma (fibroepithelioma of Pinkus)	1	0.2
	Infundibulocystic basal cell carcinoma	1	0.2
Cutaneous T-cell lymphoma	Mycosis fungoides	86.7	52
	ENKL	1.7	1
	Peripheral T-cell lymphoma not otherwise specified	1.7	1
	Subcutaneous-panniculitis-like T-cell lymphoma	1.7	1
	Primary cutaneous CD30-positive lymphoproliferative disease	5	3
	Unspecified	3.3	2
Vascular neoplasm	Kaposi sarcoma	92.3	36
	Angiosarcoma	5.1	2
	Malignant glomus tumours	2.6	1
Cutaneous metastasis	Breast origin	43.3	13
	Colonic origin	13.3	4
	Paget’s disease	10	3
	Hepatocellular carcinoma	6.7	2
	Metastatic lymphoma	6.7	2
	Head and neck cancer	3.3	1
	Unknown cutaneous metastasis	13.3	4
	Leukaemia cutis	3.3	1
Malignant melanoma	Acral lentiginous melanoma	2	0.4
	Lentigo maligna melanoma	2	0.4
	Superficial spreading melanoma	1	0.2
	Congenital melanocytic nevus	1	0.2
	Unknown melanoma	5	0.9
	Nodular melanoma	18	3.2

## Data Availability

Data are available upon request by emailing the first author.
